# Supplementary material for: Improving Aerosol Characterization Using an Optical Particle Counter Coupled with a Quartz Crystal Microbalance with an Integrated Microheater
Source: Sensors (Basel). 2024 Apr 13;24(8):2500. doi: 10.3390/s24082500 (PMC11054716; doi:10.3390/s24082500)
Supplement: Supplementary file 1 [file sensors-24-02500-s001.zip › sensors-2936829-supplementary.pdf]

Supplementary materials

Improvement of Aerosol Characterization by OPC Coupled with a QCM with an Integrated Microheater

Emiliano Zampetti *, Maria Aurora Mancuso, Paolo Papa, Andrea Bearzotti and Antonella Macagnano

Institute of Atmospheric Pollution Research–National Research Council (IIA-CNR), Research Area of Rome 1, Strada Provinciale 35d, 9 – 00010, Montelibretti (RM)

In figure S1 we report a photograph of the setup developed to perform all the measurements presented in the study. Different solutions were nebulized using an ultrasonic piezo vibrating mesh atomizer equipped with 1100 holes, each with a diameter of 6 μm and operated at a vibrating frequency of 110 kHz by a suitable homemade controller circuit (Nebulizer control board). The nebulizer was positioned within a 2 liter chamber (Generation chamber).

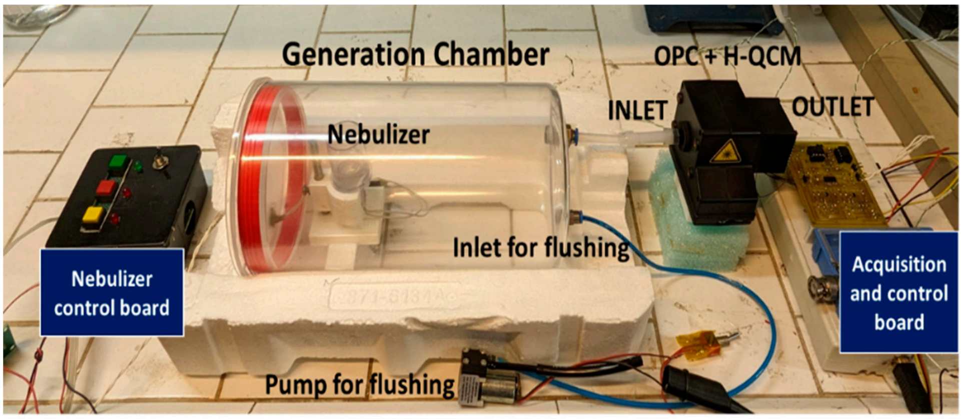

Figure S1. Measurement setup with the nebulization chamber and the coupled OPC+H-QCM system.

The OPC+H-QCM inlet was connected to the output of the generation chamber. Using the OPC integrated fan, the aerosol was propelled from the chamber and then analyzed by OPC+H-QCM system. The acquisition board comprised dual oscillator circuits designed to compensate possible H-QCM frequency variation caused by changes in temperature. An acquisition board (by National Instrument) was employed to acquire the OPC and the H-QCM outputs signals and to transmit the data to a PC unit. Additionally, the acquisition board controlled the temperature of the integrated heater on H-QCM.

OPC is based on laser scattering measurements and it is suitable to develop portable low cost measurement device for aerosol (or PM) or air quality monitoring system. By using an integrated fan (work at controlled speed), the air sample reaches the scattering chamber, where intersects with a collimated beam of laser light source. This phenomena generates volume scattering toward the photodetector device with different angles correlated to particle dimensions. Mie scattering theory may be used to relate the scattered laser power to the aerodynamic diameter of the particle by approximating the PM as homogeneous spheres [25-28].

Figure S2 shows a detailed view of the developed sensor system. In particular, the adapter was designed to optimize the aerosol focalization onto the H-QCM active measurement area while minimizing the impedance.

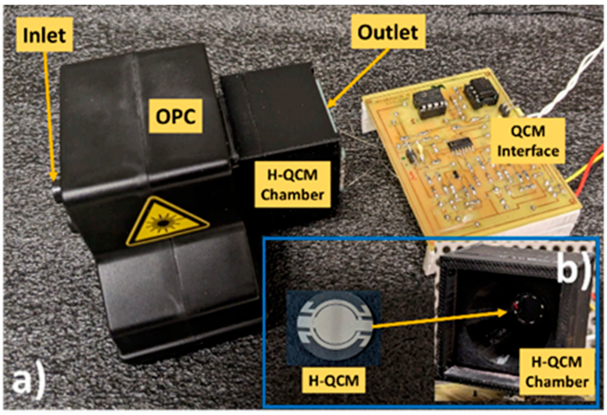

Figure S2. Photograph of the developed sensor system (a). The H-QCM was fixed by a support in the H-QCM measurement chamber (b).

Figure S3 illustrates H-QCM frequency shifts versus temperature (12 increasing steps) without deposited mass. The frequency and temperature errors were 1 Hz and 0.1 °C respectively.

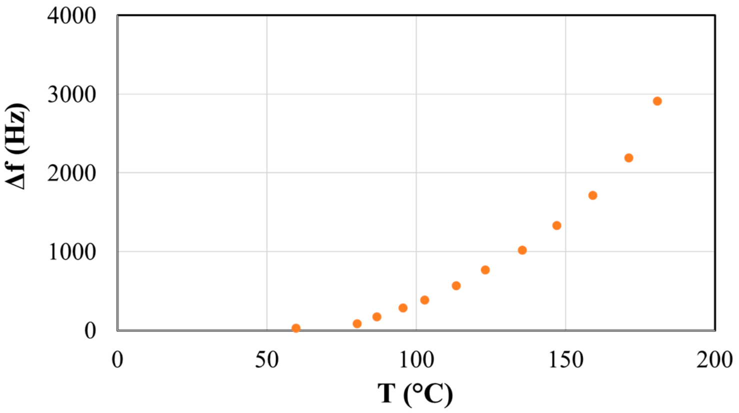

Figure S3. H-QCM frequency shifts versus temperature (12 increasing steps) without deposited mass. The frequency and temperature errors were ±1 Hz and ±0.1 °C respectively.

Figure S4 illustrates the dynamic frequency shift of the H-QCM resulting from consecutive increasing steps of temperature under air flow generated by OPC fan.

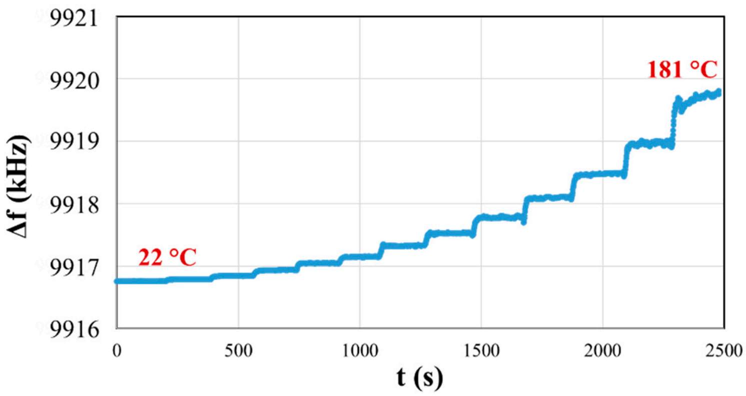

Figure S4. The H-QCM exhibited frequency variations as temperature increased, beginning from 22 °C. At the maximum temperature reached, approximately 181 °C the frequency shift was about 3000 Hz.

To prevent potential damage to the H-QCM caused by power dissipation characteristics of the thin film heater (deposited on crystal surface), operations were limited to a maximum temperature of 170 °C. This temperature was sufficient to desorb the liquid phase of aerosol samples.

Figure S5 reports an example of the counts, as a function of the aerodynamic diameter (d), of NaCl 1:10 and distilled water (H2Odist).

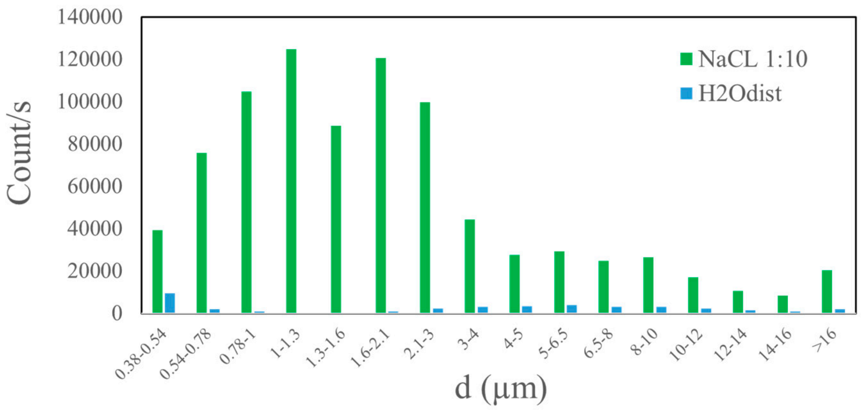

Figure S5. Counts as a function of the aerodynamic diameter (d) of NaCl 1:10 and distilled water (H2Odist).

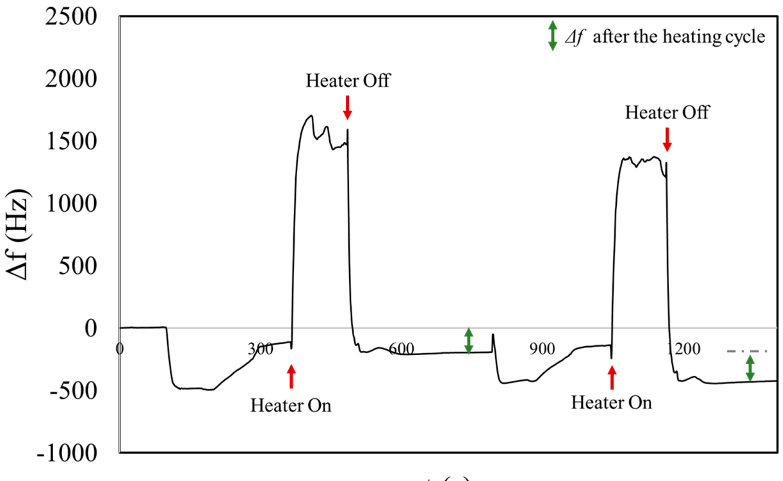

Figure S6. Chronogram of frequency shift due to two consecutive $NaCl_{1:2}$ aerosol measurements. In particular the graph highlighted the behavior before and after heating cycles.

Figure S6 shows the chronogram for the $NaCl_{1:2}$. The conditions observed are entirely analogous to those observed for the physiological solution. Before heating, both solid NaCl and liquid H_2O are present. This condition can result in surface dynamics that delay the stabilization of the signal. Once again, we can observe the dimensional effect highlighted after heating, where initially salt crystals contain hydration water. Some crystals may be larger than 2 microns in size and thus remain undetected by the 10 MHz QCM. Indeed, for

both $NaCl_{phy}$ and $NaCl_{1:2}$, the CMD calculated from the measurements obtained with the OPC (2.90 µm and 2.83 µm respectively) is consistent with this interpretation of the chronograms.

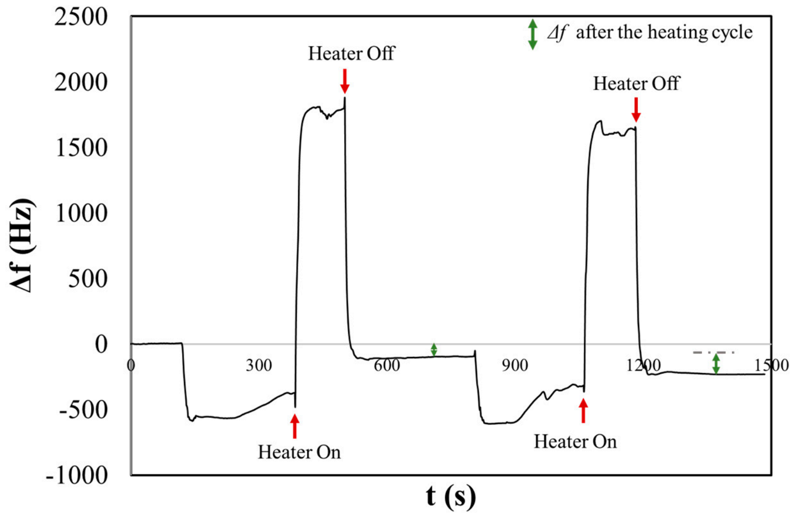

Figure S7. Chronogram of frequency shift due to two consecutive $NaCl_{1:10}$ aerosol measurements. In particular the graph highlighted the behavior before and after heating cycles.

Figure S7 illustrates the chronogram for the $NaCl_{1:10}$. In this case, the majority of salt crystals are presumably below 2 µm in size, as confirmed by measurements with the OPC, resulting in a CMD of 1.75 µm. Therefore, the decrease in Δf is attributed to water evaporation and consequently, to the loss of mass from the quartz crystal surface, as predicted by eq. 1.

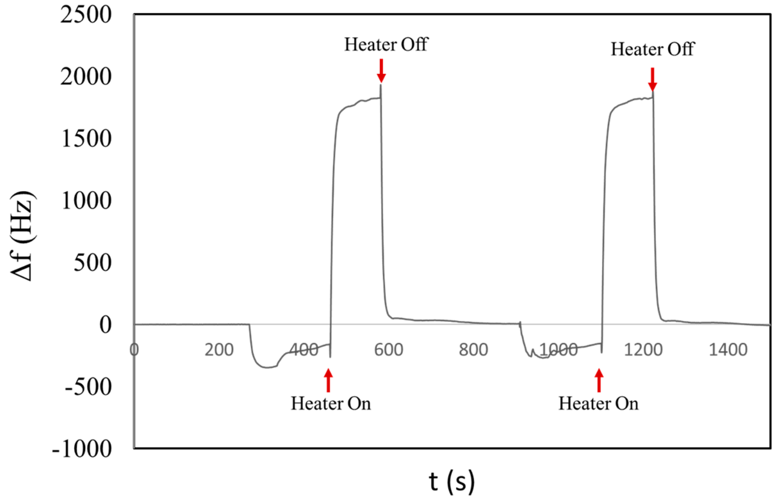

Figure S8. Chronogram of frequency shift due to two consecutive liq80:20 aerosol measurements. In particular the graph highlighted the behavior before and after heating cycles.

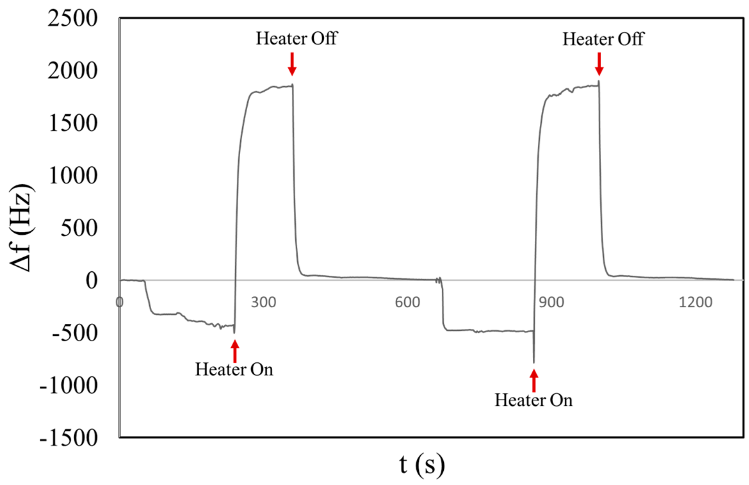

Figure S9. Chronogram of frequency shift due to two consecutive liq20:80 aerosol measurements. In particular the graph highlighted the behavior before and after heating cycles.

In both chronograms (Figure S4 and S5) related to the PG/VG liquid solutions (liq80:20 and liq20:80), following heating, the restoration of the initial frequency is observed, as also noted for the PG/VG liq50:50.
